# The effect of a distance education training program on nurse Interns' readiness for distance education and their perceptions of lifelong learning

**DOI:** 10.1002/nop2.2115

**Published:** 2024-03-07

**Authors:** Doaa El Demerdash, Lucy Ahmed Abuelela, Mimi Mohamed Mekkawy, Amany Anwar Saeed Alabdullah, Sally Mohammed Farghaly Abdelaliem

**Affiliations:** ^1^ Nursing Education Department, Faculty of Nursing Damanhour University Damanhur Egypt; ^2^ Nursing Education Department, Faculty of Nursing Galala University Egypt Egypt; ^3^ Medical Surgical Nursing Galala University ‐ Assiut University Assiut Egypt; ^4^ Department of Maternity and Child Health Nursing, College of Nursing Princess Nourah bint Abdulrahman University Riyadh Saudi Arabia; ^5^ Nursing Management and Education Department, College of Nursing Princess Nourah bint Abdulrahman University Riyadh Saudi Arabia

**Keywords:** distance education, lifelong learning, nurse interns, perceptions

## Abstract

**Aim:**

The purpose of this study is to assess the effect of a distance education training program on nurse interns' readiness for distance education and their perceptions of lifelong learning.

**Design:**

A quasi‐experimental research approach with one‐group, pre/post‐test was used.

**Methods:**

The study used a quasi‐experimental research approach and was carried out at Damanhour University's Faculty of Nursing. A study was carried out on 345 interns' students. All nursing interns enrolled in the 2020–2021 internship training year. The researchers employed a program that contained a distance education readiness assessment as well as a questionnaire about the perceived advantages of lifelong learning.

**Results:**

The majority (99.7%) of nurse interns were highly ready for distance education, whereas only 0.3 percent were moderately ready following the training program implementation immediately. In comparison to pre‐training, the majority (91.9%) of them were somewhat ready for distance education, while just 7.2 percent were highly prepared. Furthermore, the majority (97.1%) of them had high total skills of distance education after implementation of the training program by 3 months, and 95.4 percent had high total skills of learning immediately after the training program, whereas 26.1% of nurse interns had high total skills before the training program, at *p* value 0.01.

## 1 BACKGROUND

Distance education is described as “instruction and planned learning in which teaching generally happens in a distant location from learning, necessitating connection via technology as well as particular institutional organization” (Jowsey et al., 2020). Distance education is a relatively new kind of instruction that necessitates the use of modern technology. It is a popular format in educational settings because of its adaptability to students' requirements. It offers teaching and learning over long distances by utilizing some tools. Distance education has been the sole alternative to higher education to improve access for nurses who are unable to attend regular lectures (Tümen Akyildiz, 2020). The terms ‘distance learning’ and ‘distributed learning’ are sometimes used interchangeably because ‘distance learning’ suggests that there is a central institution from which the students are remote, while ‘distributed learning’ means that both the students and the institution are dispersed. That is, there are educators, offices, and places near the students who are part of the medical school in dispersed learning (WFME, 2021).

## 2 INTRODUCTION

Higher education institutions utilize technology in various ways, but successful integration requires educators' acceptance. The goal is to promote inclusivity, drive innovation, and enhance efficiency, with technology being used in teaching techniques (James, 2014; Katz & Macklin, 2009). The use of technology in today's health education programs is a crucial learning opportunity for students, as it will first improve future health care professionals' preparation and, second, enable the development of professional skills that will prepare them for future job environments. Technological advancements have led to the development of distance education, which enables training at various times and locations without physical participation. This space‐free approach has gained popularity and is now the fundamental design rationale in modern educational procedures, allowing individuals and collective structures to train at any time and location (Cousins et al., 2022; Nsouli & Vlachopoulos, 2021; Van Rensburg, 2018).

Nursing interns, experienced adult learners, are actively pursuing continuing professional education to expand knowledge and learn in the changing global market. They are self‐directed, highly motivated, and driven by career advancement, employment stability, upward mobility, and personal motivations (Hossain et al., 2020). They understand why and what they need to study (Wei & Chou, 2020). Adult learners, diverse in educational backgrounds and ambitions, often reflect on their experiences, influenced by their daily duties and varying expectations (Curran et al., 2019). As a result, there is a need for an educational environment in which adult learners may select their own educational processes, freely communicate their thoughts, and maintain their educational process alongside their personal life (Kara et al., 2019).

Distance education offers flexibility for nursing interns, allowing them to control their learning processes. To ensure learner‐centered design, programs should address various learners' needs, including those of adults. Understanding the relationship between learners' characteristics and distance education environments is crucial for effective experiences (Dilmaç, 2020). Through distance education environments, learners may control learning processes anywhere and whenever they choose. Furthermore, they can participate in greater engagement using the tools (e.g., discussion and chat) provided by distance education environments (Sailsman, 2020). Distance education offers learners the ability to acquire virtual teaming skills, control their learning processes, and receive personalized training. It also provides life‐long learning opportunities, supporting professional development programs zzcrucial for nurses' knowledge retention. This is a significant advantage for nurses in their professional development (Kara et al., 2019).

### 2.1 Study significance

There is a global need to expand the number of graduates, particularly graduates in health sciences, because there is a global shortfall of 7.2 million healthcare professionals (Katz & Macklin, 2009). The growing demand for graduates necessitates increased accessibility to education. Distance education can be overwhelming for students with limited computer literacy and internet connectivity issues. Successful assistance may be provided by determining the best remote education approach (James, 2014). From the perspective of nursing interns, the benefits of online learning include greater student satisfaction, motivation, problem‐solving abilities, and higher‐order thinking capabilities. The increasing participation of nursing interns in distance learning is driven by lifelong learning, equal access to courses worldwide, promotion, and flexibility. This growth necessitates increased attention to ensure the effectiveness of distance education programs and retention (Van Rensburg, 2018). Because of its flexibility, distance education obviously offers learners the advantage of life‐long learning.

Lifelong learners are in high demand in both the domestic and foreign labor markets. Furthermore, in order to become successful learners, lifelong learners require adequate advice, counselling, and support at all ages and stages of life and employment (Hossain et al., 2020). Lifelong learners are optimistic, curious, and enthusiastic about learning, relying on failures to understand what doesn't work. They are constantly aware of change and variation, utilizing their skills to question, reason, and assess (Wei & Chou, 2020).

Healthcare providers must regularly update their skills through lifelong learning, allowing for skill renewal and upgrading in healthcare settings. However, there is limited understanding about nurses' experiences and perceptions of lifelong learning, and there is no comprehensive global picture of how nurses interpret and experience it (Zhou et al., 2020). Students' readiness for distance education refers to their ability to execute learning activities through distance or in an online context (Roller‐Wirnsberger et al., 2019). Technology‐mediated distance education offers cost‐effective, adaptable, and extensive resources beyond the classroom, making it crucial for nursing education due to students' clinical placements (Moore, 2020).

There is a lack of studies in this field, as in some quantitative studies, they focused on the satisfaction of nursing students with e‐learning and the experiences of students, managers, and educators have been investigated, but less experimental studies have been done in this area (Farsi et al., 2020, 2022). So, the current study is among the first to investigate the effect of the distance education training program on intern nurses' readiness for distance education and assess their perception of lifelong learning in Egypt. Hence, there is an urgent need to investigate aspects including perception and preparation for distance education, as well as the perceived benefits of lifelong learning. The researchers aimed to assist learners in acquiring 21st century abilities and spark long‐term educational growth. To achieve these objectives, embedded teaching programs must take on the responsibility for change in order to provide their learners with these skills and competencies. Therefore, the following hypotheses of the study were postulated:

*H1*: Nurse interns' readiness for distance education will increase with their participation in the distance education training program.
*H2*: The distance education training program will have a significant effect on nurse interns' perceptions of lifelong learning.

## 3 METHODS

Aim: the study aimed to assess the effect of distance education training program on nurse interns' readiness for distance education and their perceptions of lifelong learning.

### 3.1 Research design

In this work, a quasi‐experimental research approach with one‐group, pre/post‐test was used.

### 3.2 Setting

The study was carried out in the Faculty of Nursing, Damanhour University.

### 3.3 Subjects and sampling

**FIGURE 1:**
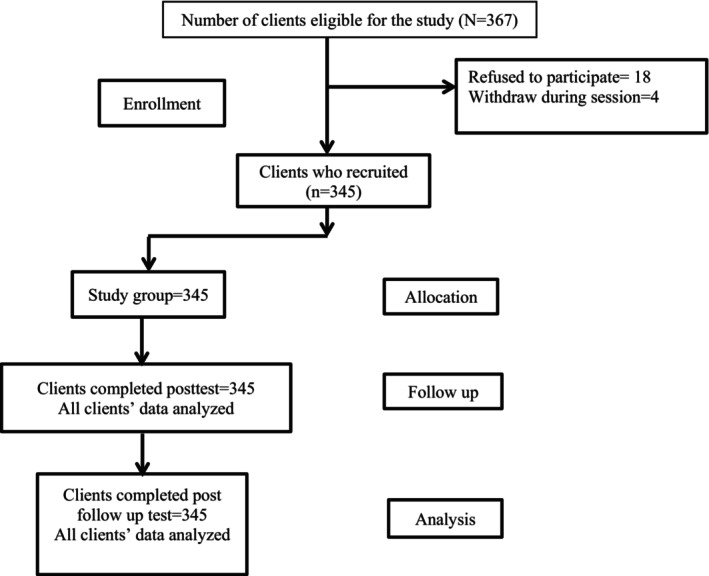
Consolidated standards of reporting Quasi‐experimental study (CONSORT).

A non‐probability sampling technique was applied using convenience sampling of 367 nursing internship students in the internship year 2020–2021 were recruited from one study setting in Egypt. Of them, 345 (96.6%) agreed to participate in the study and completed the self‐administered questionnaire after the training program. The inclusion criteria were the nursing internship students who were working in their clinical settings for not less than two months (Figure 1).

### 3.4 Tools

#### 3.4.1 Tool I: Distance education readiness questionnaire

The questionnaire was adapted and modified from Kara and Can (2019), Aleshkovskiy et al. (2020), and Bolliger and Halupa (2018) dimensions of distance education after doing a thorough analysis of the literature to identify whether a student has a high, moderate, or low degree of preparation for distance education ^(13,21,22)^. It comprised two sections:

*1st section: Section I*: personal data as: 5 questions on personal data: gender, age, English fluency, availability of private work in addition to the following aspects: internship, grade point average (GPA) of the undergraduate program, distance education literacy profile as university training distance and/or distributed/online courses, number of received university training courses, self‐training online courses, received self‐training online courses, personal IT access, and personal internet access.

*2nd Section*: Distance Education Preparation; this section included 24 statements and four subscales of skills (online student qualities, time management, communication, and technical) that assessed student readiness for distance education. Responses were graded on a four‐point scale: strongly agree, agree, disagree, and strongly disagree. Reverse scores were assigned to statements 10, 15, and 21, for example; “I enjoy working face‐to‐face with other students in groups”. The higher the mean score, the higher the readiness for distance education, as the overall readiness score is distributed as follows: low readiness if the score is 24 to 48, moderate readiness if the score is 49 to 67, and high readiness if the score is 68 to 96.

#### 3.4.2 Tool II: Perceived benefits of lifelong learning

The researchers developed this tool after doing a comprehensive assessment of the literature ^(10,12,14,16–18,23)^. It composed of two sections:

*1st Section*: baseline knowledge on the concept of lifelong learning and, benefits of lifelong learning.

*2nd Section*: Perceived skills earned by lifelong learning; this section is divided into 5 subscales: curiosity (5 items), openness to learning (8 items), access to information and information literacy (4 items), self‐direction and self‐evaluation (6 items), and sustainability in lifelong learning (2 items). The higher the mean score, the higher the perception for lifelong learning, as the overall perception score is distributed as follows: low perception if the score is 25 to 49, moderate if the score is 50 to 70, and high perception if the score is 71 to 100.

### 3.5 Study tools adaptation, validity, and reliability

#### 3.5.1 Tools adaptation

**FIGURE 2:**
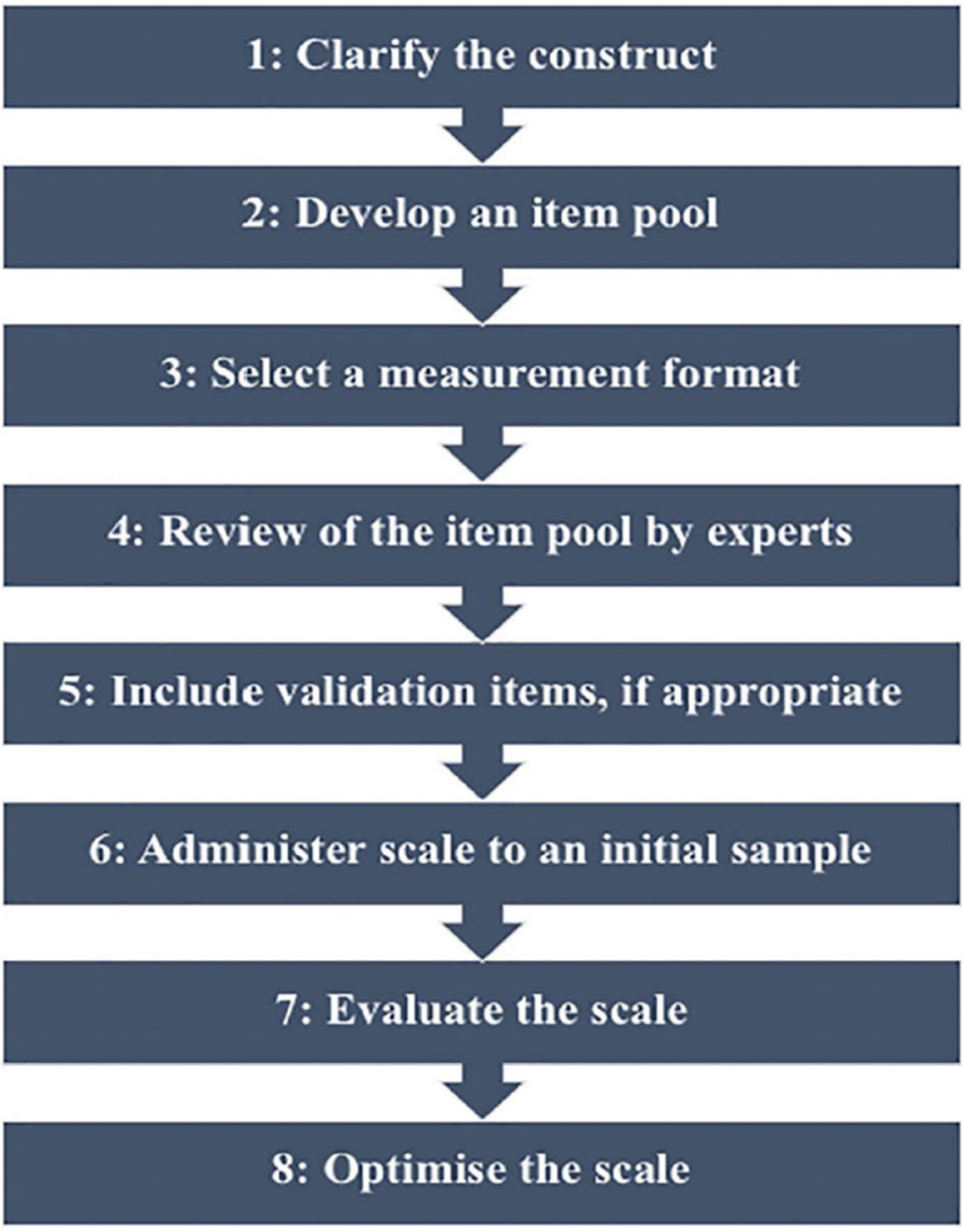
Steps in scale development (DeVellis, 2017).

Since all participants were university graduates with English proficiency, questionnaires were constructed in the English language. We employed various methods to evaluate their validity and reliability, such as following the Steps 1–6 of DeVellis' (2017) model for scale development (see Figure 2) in relation to the coding instrument created. Clarification of the construct and development of an item pool were informed by a review of the literature review (Nifadkar et al., 2019; Thuan & Thanh, 2020 **)**. In addition, content validity, exploratory factor analysis (EFA), confirmatory factor analysis (CFA), corrected item‐total correlations, and Cronbach's alpha were applied. We used IBM SPSS software package version 22.0 and AMOS version 23.0 for the analyses.

#### 3.5.2 Content validity

A group of seven academics from the discipline, including three professors of information and data management and four professors of nursing administration. The panel identified word choice problems, typing errors, and punctuation errors. Several words were changed in response to their suggestions. In order to verify the accuracy and functionality of the instruments and determine the amount of time needed to complete study questionnaires, a pilot study with 35 nursing interns of the previous year to investigate the knowledge questionnaire's stability over time and showed a high positive significant correlation (r ranged from 0.782 to 0.862). The study sample did not contain the pilot sample.

#### 3.5.3 Construct validity

Both exploratory factor analysis (EFA) and confirmatory factor analysis (CFA) were used to assess the construct validity of the developed tools.

Kaiser normalization was used in conjunction with Promax rotation to conduct an exploratory factor analysis (EFA). The objective of the EFA was to pinpoint the fundamental elements or dimensions that account for the variation in each questionnaire item's response.

The EFA of the distance education readiness questionnaire revealed a clear and consistent factor structure that reflected the two dimensions of the scale with high boldface loadings for all items, ranging from 0.544 to 0.943 which means that the items strongly contribute to their factor. The Kaiser‐Meyer‐Olkin (KMO) measure of sampling adequacy was 0.814, which indicates the appropriateness of the data for factor analysis and that there is a high degree of common variance among the items.

The EFA of the perceived benefits of lifelong learning questionnaire using Kaiser normalization revealed that there are two dominant sections that accounts for most of the variance in the responses. These factors have high loadings for all items, ranging from 0.634 to 0.745. This implies that it represents a general dimension of polychronic‐monochronic tendency, and that all items measure the same construct. The KMO value is 0.856 for section and 0.871 for section 2, indicates that the data are very suitable for factor analysis, and that there is a high degree of common variance among the items.

#### 3.5.4 Confirmatory factor analysis

CFA was done using structural equation modelling (SEM) for the mean and standard deviation of the two scales used in the study (distance education readiness, and perceived benefits of lifelong learning). The CFA aimed to test the fit of the factor structure derived from the EFA to the data. The CFA confirmed that the model fit the data very well (comparative fit index [CFI] = 1.000, incremental fit index (IFI) = 1.000, root mean square error of approximation (RMSEA) = 0.117, Model *X* ^2^ = 5.120, *p* = 0.001*).

### 3.6 Reliability

*The reliability of the tools was assessed using the* corrected item‐total correlations and internal consistency.

*The corrected item‐total correlations* is the correlation between an item and the total score of the scale, excluding that item. It is a measure of how well each item fits with the overall scale. The corrected item‐total correlations of the distance education readiness questionnaire showed that all items were positively and significantly related to their respective sections and to the overall score of the survey, ranging from 0.567 to 0.987. The corrected item‐total correlations of the perceived benefits of lifelong learning scale showed that all items were positively and significantly related to the scale, ranging from 0.682 to 0.736. This means that all items are positively related to their respective subscales, and that they measure the same construct as the other items in the subscale.

*Internal consistency*, the Cronbach's alpha was used to measure the internal consistency of the tools. It is a measure of how well a set of items measures a single construct or dimension. It ranges from 0 to 1, with higher values indicating higher reliability and internal consistency. The Cronbach's alpha values for the distance education readiness questionnaire are 0.814 for the overall survey. The Cronbach's alpha values for all subscales perceived benefits of lifelong learning scale were 0.864. These results indicate acceptable reliability and internal consistency of the study tools.

### 3.7 Statistical analysis

Data were fed to the statistical package of social science (SPSS, version 24). Socio‐demographic characteristics presented with frequencies and percentages. Mean and standard deviation (SD) describe the studied variables. The readiness for distance education and the knowledge pre and post training program implementation were tested using Friedman test to address H1. *X* ^2^ Chi square test was used to compare the pre‐test/post‐test levels and its relation with the sociodemographic. Pearson correlation coefficient analysis (*r*) was utilized to test the relationship among variables to address H2. All statistical analyses were applied using an alpha of 0.05. The normality of data distribution was tested as the Shapiro–Wilk test was used to determine the normality of the data.

### 3.8 Fieldwork and data collection

Written approval was obtained from the identified faculty administration for data collection. The study was executed over two main phases: pre‐ and post‐intervention phases. The pre‐intervention phase involved a pre‐test and content development for distance education training sessions, while the post‐intervention phase included a post‐test (two times, immediately after the training program and after 3 months of the training program) and data analysis. Study participants were required to respond to questionnaires concerning distance education and lifelong learning before and after the intervention. During the pre‐intervention phase, the nurse interns introduced the pre‐test questionnaires to assess their knowledge of the study variables before implementing the training sessions. The researchers prepared the content and handout based on reviewing the related literature and participants' learning needs of pre‐test results. A handout was revised by academic experts and distributed among nurse interns. Arrangements for sessions' time and place were decided with the nurse interns according to their work schedules and workloads. (Table 1).

**TABLE 1:** Study Framework.

Phases	Actions	Tools
Pre‐intervention phase	Pre‐test	Study Questionnaires
	Content development	Nurse interns' learning needs, and related literature Content reviewed by experts
	Training sessions implementation	Content: Broad and specific objectives Distance education definition and general review Relate information literacy to lifelong learning ability. Appreciate sustainability in Lifelong Learning. Apply open access map to international distance education training programs Principles, elements, techniques, benefits, and advantages of distance education Process, strategies, facilitators, and hinders/barriers The relationship between distance education and lifelong learning Reflective practice
Post‐intervention phase	Post‐test	Study Questionnaires (immediately and after 3 months)
	Data analysis	Compare pre‐test with post‐test results. Assess the effect of the training program, readiness for distance education, and level of perception of lifelong learning

During the first session, the researchers established a rapport with the nurse interns and presented the objectives of the training sessions, distance education definition, overview, principles, elements, benefits, techniques, and advantages. Also, relate information literacy to lifelong learning ability, sustainability in lifelong learning, and applying open access map to international distance education training programs. The researchers explained the distance education process, facilitators, and hinders/barriers for execution in the educational institutions within the second session. In the third session, the relationship between distance education and lifelong learning was discussed besides getting participants' feedback and reflection on the sessions. The content was presented online through Microsoft team using appropriate and collaborative teaching strategies (brainstorming, lecture, video presentation, and small group discussion). The researchers implemented 12 sessions to embrace all content to all participants. Most of the sessions were implemented in the morning duty, and few sessions were implemented in the evening shift. During the post‐intervention phase, the post‐test questionnaires were introduced instantly after the third session for each group to assess the changes in nurse interns' knowledge of distance education and lifelong learning. Every participant took around 30 min to complete the questionnaires by sending the link of the questionnaires on the official university e‐mail of the nurse interns. The duration of the study was four months in total for intervention and data collection, 2021.

### 3.9 Ethical considerations

The researchers acquired ethical approval to perform the study from The Nursing Ethics Committee at Damanhour University's Faculty of Nursing (N: 63‐2022). Following an explanation of the research aims and methods, each student participating in the nursing internship year provided individual informed permission. Students were informed that participation is entirely optional, that refusals or withdrawals have no ramifications, and that the information would be kept secret and would not influence their academic progress in the internship program.

## 4 RESULTS

Table 2 clarified that the mean age of the studied nursing interns' is 22.66 with SD 0.865. Most (80.9%) of them were female while, only 19.1% of them were male. Regarding English fluency, more than half (54.8%) of them were very good at English while less than one‐fifth (19.7%) of them were excellent. Moreover, nearly one‐fifth (22%) of them had a private work. Concerning grade point average (GPA), more than half (55.4%) of them were very good one‐third (33.3%) of them were excellent. Also, more than three quarter (76.1%) of them received one university training course and 15.8% of them had two while only 8.1% of them had three courses. It also reveals that only 29.0% of then had self‐training online courses and 36.0% of them had nursing procedure self‐training online course. Furthermore, more than three quarter (75.7%) of them had personal IT access, and the vast majority (91.6%) of them had personal internet access.

**TABLE 2:** Demographics of nursing interns according to demographic data (*n* = 345).

Items		Total (*N* = 345)	
		No.	%
Age (years)	21‐	14	4.1
	22‐	152	44.1
	23‐	131	38.0
	24‐25	48	13.9
	(Mean ± SD)	22.66 ± 0.865	
Gender	Male	66	19.1
	Female	279	80.9
English fluency	Bad	2	0.6
	Fair	5	1.4
	Good	81	23.5
	Very good	189	54.8
	Excellent	68	19.7
Private work	No	269	78.0
	Yes	76	22.0
Grade point average (GPA)	Good	39	11.3
	Very good	191	55.4
	Excellent	115	33.3
University training online courses	No	60	17.4
	Yes	285	82.6
Number of received university training courses		***N* = 285**	
	One	217	76.1
	Two	45	15.8
	Three and more	23	8.1
Self‐training online courses		***N* = 345**	
	No	245	71.0
	Yes	100	29.0
Received self‐training online courses		***N* = 100**	
	Nursing procedure	36	36.0
	Language	25	25.0
	Human resources	4	4.0
	Computer	18	18.0
	Infection control	15	15.0
	Quality management	24	24.0
	COVID‐19	7	7.0
Have personal information technology (IT) access		***N* = 345**	
	No	84	24.3
	Yes	261	75.7
Have personal internet access		***N* = 345**	
	No	29	8.4
	Yes	316	91.6

The findings reveal that there is a highly significance difference regarding nursing interns' readiness to distance education pre and post‐intervention (*p* value <0.01). Moreover, the table shows that all (100%) studied nursing interns had high readiness to distance education post‐intervention (after 3 months of the intervention). Also, the majority (99.7%) of them had high readiness to distance education, while only 0.3% of them had moderate readiness post‐intervention (immediately after the intervention). Compared with pre intervention readiness, the majority (91.9%) of them had moderate readiness to distance education while only 7.2% of them had high readiness. (Table 3).

**TABLE 3:** Nursing interns' level of readiness for distance education (pre‐ and post‐intervention).

Items	Pre intervention (*N* = 345)		Post‐intervention (immediately) (*N* = 345)		Post‐intervention (after 3 months) (*N* = 345)		Friedman test *p* value
	No.	%	No.	%	No.	%	
Readiness to distance learning							
Low	3	0.9	0	0.0	0	0.0	28.766 *p* < 0.01**
Moderate	317	91.9	1	0.3	0	0.0	
High	25	7.2	344	99.7	345	100.0	

** Highly significant at *p* < 0.01.

Furthermore, there is a highly significance difference regarding studied nursing interns' perception of lifelong learning pre and post‐intervention (*p* value <0.01). Regarding benefits of online learning, the majority (96.5%) of them had high total benefits post‐intervention (immediately), and the majority (92.2%) of them had high total benefits post‐interventions (after 3 months) compared with pre intervention only 28.1% of them had high total benefits pre intervention at *p* value <0.01. Moreover, the majority (97.1%) of them had high total skills of online learning post‐intervention (after 3 months) and 95.4 of them had high total skills learning post‐intervention (immediately), while only 26.1% of them had high total skills pre intervention, at *p* value <0.01. (Table 4).

**TABLE 4:** Nursing interns' level of perception of lifelong learning (pre‐ and post‐intervention).

Items	Pre intervention (*N* = 345)		Post‐intervention (immediately) (*N* = 345)		Post‐intervention (after 3 months) (*N* = 345)		Friedman test *p* value
	No.	%	No.	%	No.	%	
Benefits of lifelong learning							
Concept of online learning							
Low	2	0.6	0	0.0	0	0.0	25.078 *p* < 0.01**
Moderate	216	62.6	4	1.2	46	13.3	
High	127	36.8	341	98.8	299	86.7	
Benefits of online learning							
Low	17	4.9	3	0.9	0	0.0	27.081 *p* < 0.01**
Moderate	220	63.8	25	7.2	19	5.5	
High	108	31.3	317	91.9	326	94.5	
Total benefits of online learning							
Low	4	1.2	0	0.0	0	0.0	29.800 *p* < 0.01**
Moderate	244	70.7	12	3.5	27	7.8	
High	97	28.1	333	96.5	318	92.2	
Skills of lifelong learning							
Curiosity							
Low	8	2.3	0	0.0	0	0.0	22.053 *p* < 0.01**
Moderate	107	31.0	4	1.2	15	4.3	
High	230	66.7	341	98.8	330	95.7	
Openness							
Low	7	2.0	0	0.0	0	0.0	21.411 *p* < 0.01**
Moderate	178	51.6	3	0.9	43	12.5	
High	160	46.4	342	99.1	302	87.5	
Access							
Low	6	1.7	0	0.0	0	0.0	24.300 *p* < 0.01**
Moderate	269	78.0	43	12.5	32	9.3	
High	70	20.3	302	87.5	313	90.7	
Self‐direction							
Low	6	1.7	0	0.0	0	0.0	25.732 *p* < 0.01**
Moderate	269	78.0	43	12.5	30	8.7	
High	70	20.3	302	87.5	315	91.3	
Sustainability							
Low	48	13.9	2	0.6	0	0.0	24.017 *p* < 0.01**
Moderate	237	68.7	6	1.7	10	2.9	
High	60	17.4	337	97.7	335	97.1	
Total Skills of online learning							
Low	10	2.9	0	0.0	0	0.0	30.125 *p* < 0.01**
Moderate	245	71.0	16	4.6	10	2.9	
High	90	26.1	329	95.4	335	97.1	
Total							
Low	9	2.6	0	0.0	0	0.0	34.612 *p* < 0.01**
Moderate	249	72.2	11	3.2	0	0.0	
High	87	25.2	334	96.8	345	100.0	

** Highly significant at *p* < 0.01.

Moreover, there is a positive correlation between studied nursing interns' readiness for distance education Pre intervention and their perception of lifelong learning pre intervention (*p* < 0.01). Also, there is a positive correlation between studied nursing interns' readiness for distance education post‐intervention (immediately) and their perception of lifelong learning post‐intervention (immediately) (*p* < 0.01). Furthermore, there is a positive correlation between studied nursing interns' readiness for distance education post‐intervention (after 3 months) and their perception of lifelong learning post‐intervention (after 3 months) (*p* < 0.01). (Table 5).

**TABLE 5:** Correlation between the studied nursing interns' mean score of perception and readiness of online learning pre‐ and post‐intervention.

Study variables	*r*	*p* value
Readiness for distance education and perception of lifelong learning (*pre intervention*)	0.487	*p* < 0.01^a^
Readiness for distance education and perception of lifelong learning (*immediately post‐intervention*)	0.574	*p* < 0.01^a^
Readiness for distance education and perception of lifelong learning (*3 months post‐intervention*)	0.439	*p* < 0.01^a^

*Note*: *R*, Pearson correlation.

^a^ High significant at *p* < 0.01.

## 5 DISCUSSION

The current study examined the effect of distance education training program on nurse interns' readiness for distance education and their perceptions of lifelong learning. The results verified that there is significant improvement in nursing interns' readiness for distance education, as evidenced by the fact that all of the nursing interns exhibited high readiness to online learning after the post‐intervention (after 3 months of the training program). In addition, following the initial session, the great majority of them demonstrated a high level of readiness for distance education. Only a fraction of them exhibited great readiness compared to pre‐intervention readiness. These findings were linked to the success of the training program offered by the researcher, which was based on the requirements of nursing interns at the time of pre‐assessment. These findings are backed by Liu (2019) study, which employed a one‐group pre‐test–post‐test approach, 445 pre‐test and 624 post‐test datasets, and discovered that the independent samples t‐test results revealed statistically significant increase in student readiness abilities for distance education. Tsai, 2020 also discovered that competency‐based learning has a good influence on learning readiness and distance education experience. Furthermore, Pete et al., 2017 demonstrated that, while students' psychological readiness for e‐Learning was strong, they lacked technological and equipment readiness. Furthermore, Şenyuva and Kaya (2014) conducted research on 162 second‐grade children in Turkey and discovered that the difference in students' readiness for self‐directed learning before and after the web‐based course was statistically and highly significant.

In terms of perspective of lifelong learning, the current study found that nursing interns' perceptions of the benefits of distance education and lifelong learning skills increased immediately after the training program and after 3 months of its implementation. These findings might be attributed to the researchers' use of effective teaching approaches to allow access to knowledge and the usage of commonly used phrases, as well as to provide a chance for questioning. The results go in line with Kim et al., 2021 who conducted a quasi‐experimental design on a sample of 164 senior nursing students and discovered a substantial gain in understanding of distance education and learning flow in the post‐test. Furthermore, according to Rouleau et al., 2019, the impacts of e‐learning are mostly recorded in terms of nurse responses, knowledge, and abilities (ie, the first two levels of the Kirkpatrick model). The usefulness of e‐learning interventions for nurses in a continuing education environment in terms of how the learning may be transferred to modify practice and influence patient outcomes is uncertain. Swaminathan et al. (2021) conducted a study on 158 entry‐level nursing graduate students in Chennai, India, and found that the majority of them are confident in accessing internet information. Willingness to study online, prior experience with distance education, and perceived benefits of the online component may all impact a learner's readiness for lifelong learning.

There was a significant difference between the examined nursing interns' readiness for distance education before and after the training program. These findings are consistent with Herguner et al. (2020), who conducted a study on 306 university students and discovered that learners' distance education attitudes had a beneficial influence on their readiness for lifelong learning and continuous education. According to Diab and Elgahsh (2020), there is a large statistically significant negative link between the challenges that nursing students face and their view of e‐learning. Furthermore, Mallı et al. (2021) discovered a strong positive relationship between students' perceptions and their readiness for e‐learning. In addition, Kumar, 2021 ^(33)^ conducted a study on 155 students and found that learning readiness had a favourable influence on student satisfaction.

### 5.1 Limitation of the study

The sample for this study was confined to one institution and one pre‐test and post‐test, therefore generalizability of the results is limited. Also, the current results are based on self‐reported data, and thus, they are at risk of response bias and subjectivity. Despite this constraint, the findings of this study have implications for practice in the design of online learning and training that related assessment processes. Future longitudinal, experimental including intervention and control group, and multi‐site studies may help to overcome these limitations.

## 6 CONCLUSION

The current study investigated the effect of distance education training program on nurse interns' readiness for distance education and their perceptions of lifelong learning. The results revealed that there was a significant improvement in nursing interns' readiness to distance education following the training program. In addition, the current study found that nursing interns' perceptions of the benefits of distance education and lifelong learning skills increased after the implementation of the training program immediately and after 3 months of its implementation. Furthermore, there was a significant correlation between the readiness for distance education of the nursing interns and their readiness for lifelong learning.

## 7 IMPLICATIONS OF THE STUDY

The recent study emphasized on the need for incorporating research findings into nursing school curriculum and teaching methods. Provide a training program for faculty members on long‐term learning and how to use it in nursing sciences.

## 8 FUTURE RESEARCH

Further investigation of the aspects influencing undergraduate university students' lifetime learning. Further study on the same problem, but with a new context and a large number of people, is needed to generalize the results.

## AUTHOR CONTRIBUTIONS

All authors participated in the research idea, conceptualization, data collection, analysis and preparation of the manuscript for publication.

## FUNDING INFORMATION

The research was funded by Princess Nourah bint Abdulrahman University Researchers Supporting Project number (PNURSP2024R444), Princess Nourah bint Abdulrahman University, Riyadh, Saudi Arabia.

## CONFLICT OF INTEREST STATEMENT

The authors have no conflicts of interest to disclose.

## ETHICAL APPROVAL

The researchers acquired ethical authorization to perform the study from The Nursing Ethics Committee at X University's Faculty of Nursing (# 63–2022). Following an explanation of the research aims and methods, each student participating in the nursing internship year provided individual informed permission. Students were informed that participation is entirely optional, that refusals or withdrawals have no ramifications, and that the information would be kept secret and would not influence their academic progress in the internship program. All methods were carried out in accordance with relevant guidelines and regulations.

## INFORMED CONSENT

Informed consent obtained from all the participants included in the study.

## Data Availability

The datasets generated and/or analysed during the current study are not publicly available due to data privacy but are available from the corresponding author on reasonable request.

## References

[nop22115-bib-0001] Aleshkovskiy, I. A. , Gasparishvili, A. T. , Krukhmaleva, O. V. , Narbut, N. P. , & Savina, N. E. (2020). Russian university students about distance learning: Assessments and opportunities. Vysshee Obrazovanie v Rossii=Higher Education in Russia, 29(10), 86–100.

[nop22115-bib-0002] Bolliger, D. U. , & Halupa, C. (2018). Online student perceptions of engagement, transactional distance, and outcomes. Distance Education, 39(3), 299–316.

[nop22115-bib-0003] Cousins, E. , Preston, N. , Doherty, J. , Varey, S. , Harding, A. , McCann, A. , Harrison Dening, K. , Finucane, A. , Carter, G. , Mitchell, G. , & Brazil, K. (2022). Implementing and evaluating online advance care planning training in UK nursing homes during COVID‐19: Findings from the necessary discussions multi‐site case study project. BMC Geriatrics, 22, 419. 10.1186/s12877-022-03099-z 35562712 PMC9098790

[nop22115-bib-0004] Curran, V. , Gustafson, D. L. , Simmons, K. , Lannon, H. , Wang, C. , Garmsiri, M. , Lisa, F. , Lyle, W. , & Wetsch, L. (2019). Adult learners' perceptions of self‐directed learning and digital technology usage in continuing professional education: An update for the digital age. Journal of Adult and Continuing Education, 25(1), 74–93.

[nop22115-bib-0005] DeVellis, R. F. (2017). Scale development: Theory and applications (4th ed.). Sage Publications.

[nop22115-bib-0006] Diab, G. M. A. E. H. , & Elgahsh, N. F. (2020). E‐learning during COVID‐19 pandemic: Obstacles faced nursing students and its effect on their attitudes while applying it. American Journal of Nursing, 9(4), 300–314.

[nop22115-bib-0007] Dilmaç, S. (2020). Students' opinions about the distance education to art and design courses in the pandemic process. World Journal of Education, 10(3), 113–126.

[nop22115-bib-0008] Farsi, Z. , Afaghi, E. , Fournier, A. , Ahmadi, Y. , Sajadi, S. A. , & Aliyari, S. H. (2022). Investigating nursing Students' satisfaction with the quality of courses and virtual learning during the COVID‐19 pandemic in 2020‐2021. Turkish Online Journal of Distance Education, 23(3), 103–117.

[nop22115-bib-0009] Farsi, Z. , Aliyari, S. , Ahmadi, Y. , Afaghi, E. , & Sajadi, S. A. (2020). Satisfaction of the quality of education and virtual education during the Covid‐19 pandemic in nursing students of Aja University of medical sciences in 2020. Journal of Military Medicine, 23(2), 174–185.

[nop22115-bib-0010] Herguner, G. , Son, S. B. , Herguner Son, S. , & Donmez, A. (2020). The effect of online learning attitudes of university students on their online learning readiness. Turkish Online Journal of Educational Technology‐TOJET, 19(4), 102–110.

[nop22115-bib-0011] Hossain, M. N. , Talukder, M. S. , Khayer, A. , & Bao, Y. (2020). Investigating the factors driving adult learners' continuous intention to use M‐learning application: A fuzzy‐set analysis. Journal of Research in Innovative Teaching & Learning, 14(2), 245–270.

[nop22115-bib-0012] James, R. (2014). ICT's participatory potential in higher education collaborations: Reality or just talk. British Journal of Educational Technology, 45(4), 557–570.

[nop22115-bib-0013] Jowsey, T. , Foster, G. , Cooper‐Ioelu, P. , & Jacobs, S. (2020). Blended learning via distance in pre‐registration nursing education: A scoping review. Nurse Education in Practice, 44, 102775. 32247200 10.1016/j.nepr.2020.102775 PMC7195119

[nop22115-bib-0014] Kara, M. , & Can, G. (2019). Master's students' perceptions and expectations of good tutors and advisors in distance education. The International Review of Research in Open and Distance Learning, 20(2), 5–22.

[nop22115-bib-0015] Kara, M. , Erdogdu, F. , Kokoç, M. , & Cagiltay, K. (2019). Challenges faced by adult learners in online distance education: A literature review. Open Praxis, 11(1), 5–22.

[nop22115-bib-0016] Katz, I. , & Macklin, A. (2009). Information and communication technology (ICT) literacy: Integration and assessment in higher education. Journal of Systemics Cybernetics and Informatics, 5(4), 50–55.

[nop22115-bib-0017] Kim, S. Y. , Kim, S. J. , & Lee, S. H. (2021). Effects of online learning on nursing students in South Korea during COVID‐19. International Journal of Environmental Research and Public Health, 18(16), 8506. 10.3390/ijerph18168506 34444257 PMC8394981

[nop22115-bib-0018] Kumar, S. P. (2021). Impact of online learning readiness on students satisfaction in higher educational institutions. Journal of Engineering Education Transformations, 34(Special Issue), 64–70.

[nop22115-bib-0019] Liu, J. C. (2019). Evaluating online learning orientation design with a readiness scale. Online Learning, 23(4), 42–61.

[nop22115-bib-0020] Mallı, A. H. M. E. T. , Ekinci, H. A. S. A. N. , Seçer, E. M. R. A. H. , Demirel, N. , & Şam, C. (2021). Investigation of readiness and expectations of students of sports science faculties regarding the E‐learning process and their SelfEfficacy perceptions. Pakistan Journal of Medical and Health Sciences, 15(10), 3211–3216.

[nop22115-bib-0022] Moore, R. L. (2020). Developing lifelong learning with heutagogy: Contexts, critiques, and challenges. Distance Education, 41(3), 381–401.

[nop22115-bib-0023] Nifadkar, S. S. , Wu, W. , & Gu, Q. (2019). Supervisors' work‐related and nonwork information sharing: Integrating research on information sharing, information seeking, and trust using self‐disclosure theory. Personnel Psychology, 72(2), 241–269.

[nop22115-bib-0024] Nsouli, R. , & Vlachopoulos, D. (2021). Attitudes of nursing faculty members toward technology and e‐learning in Lebanon. BMC Nursing, 20, 116. 10.1186/s12912-021-00638-8 34193112 PMC8247129

[nop22115-bib-0025] Pete, M. , Coopasami, M. , & Knight, S. (2017). E‐learning readiness amongst nursing students at the Durban University of Technology. Health Sa Gesondheid, 22(1), 300–306.

[nop22115-bib-0026] Roller‐Wirnsberger, R. , Zitta, S. , Herzog, C. , Dornan, H. , Lindner, S. , Rehatschek, H. , Hye, F. , Kolosovski, L. , Wirnsberger, G. , Corsonello, A. , Tap, L. , Kostka, T. , Guligowska, A. , Mattace‐Raso, F. , Gil, P. , Guardado Fuentes, L. , Artzi‐Medvedik, R. , Yehoshua, I. , Formiga, F. , … Lattanzio, F. (2019). Massive open online courses (MOOCs) for long‐distance education in geriatric medicine across Europe. European Geriatric Medicine, 10(6), 989–994. 34652779 10.1007/s41999-019-00252-7

[nop22115-bib-0027] Rouleau, G. , Gagnon, M. P. , Côté, J. , Payne‐Gagnon, J. , Hudson, E. , Dubois, C. A. , & Bouix‐Picasso, J. (2019). Effects of e‐learning in a continuing education context on nursing care: Systematic review of systematic qualitative, quantitative, and mixed‐studies reviews. Journal of Medical Internet Research, 21(10), e15118. 31579016 10.2196/15118 PMC6777280

[nop22115-bib-0028] Sailsman, S. (2020). ESL students learning online: A review of literature. The Quarterly Review of Distance Education, 21(1), 45–52.

[nop22115-bib-0029] Şenyuva, E. , & Kaya, H. (2014). Effect self‐directed learning readiness of nursing students of the web based learning. Procedia‐Social and Behavioral Sciences, 152, 386–392.

[nop22115-bib-0031] Swaminathan, N. , Ravichandran, L. , Ramachandran, S. , Milanese, S. , Singaravelu, R. , & Govindaraj, P. (2021). Entry level nursing graduate students' perception and readiness toward online component of blended learning: A mixed method study. Journal of Education Health Promotion, 10(1), 163. 10.4103/jehp.jehp_771_20 34250097 PMC8249981

[nop22115-bib-0032] Thuan, L. C. , & Thanh, B. T. (2020). Leader knowledge sharing behavior and follower creativity: The role of follower acquired knowledge and prosocial motivation. Journal of Workplace Learning, 32(6), 457–471.

[nop22115-bib-0033] Tsai, C. W. (2020). Applying online competency‐based learning and design‐based learning to enhance the development of students' skills in using PowerPoint and word, self‐directed learning readiness, and experience of online learning. Universal Access in the Information Society, 19(2), 283–294.

[nop22115-bib-0034] Tümen Akyildiz, S. (2020). College Students' views on the pandemic distance education: A focus group discussion. International Journal of Technology in Education and Science, 4(4), 322–334.

[nop22115-bib-0035] Van Rensburg, E. S. J. (2018). Effective online teaching and learning practices for undergraduate health sciences students: An integrative review. International Journal of Africa Nursing Sciences, 9, 73–80.

[nop22115-bib-0036] Wei, H. C. , & Chou, C. (2020). Online learning performance and satisfaction: Do perceptions and readiness matter? Distance Education, 41(1), 48–69.

[nop22115-bib-0037] World Federation For Medical Education . (2021). Basic medical education WFME Standards for distributed and distance learning in medical education . The 2021 Revision. WFME. https://wfme.org/wp‐content/uploads/2017/05/WFME‐STANDARDS‐FOR‐DISTRIBUTED‐AND‐DISTANCE‐LEARNING‐IN‐MEDICINE_2021‐final‐1.pdf

[nop22115-bib-0038] Zhou, T. , Huang, S. , Cheng, J. , & Xiao, Y. (2020). The distance teaching practice of combined mode of massive open online course micro‐video for interns in emergency department during the COVID‐19 epidemic period. Telemedicine and e‐Health, 26(5), 584–588. 32271650 10.1089/tmj.2020.0079

